# Cutaneous vasculitis after COVID‐19 vaccination in a 41‐year‐old male

**DOI:** 10.1002/ccr3.7238

**Published:** 2023-04-22

**Authors:** Mehran Pournazari, Shirin Assar, Faraneh Farsad, Dena Mohamadzadeh

**Affiliations:** ^1^ Clinical Research Development Center Imam Reza Hospital, Kermanshah University of Medical Sciences Kermanshah Iran; ^2^ Research Centre of Loghman Hakim Hospital Shahid Beheshti University of Medical Sciences Tehran Iran

**Keywords:** COVID‐19 infection, cutaneous vasculitis, leukocytoclastic vasculitis, SARS‐CoV‐2 vaccine

## Abstract

Leukocytoclastic vasculitis could be a possible adverse event of different SARS‐CoV‐2 vaccines. Clinicians and manufacturers should be aware of this adverse event for appropriate diagnosis and treatment.

## Abstract 2

Cutaneous vasculitis has been associated with both Coronavirus disease‐2019 (COVID‐19) and SARS‐CoV‐2 vaccines. We reported a 41‐year‐old male who developed bullous lesions and erythematous plaques leading to atrophic patches 2 weeks after injection of the second dose of the COVID‐19 vaccine. The skin biopsy showed leukocytoclastic vasculitis. The disease was self‐limited and no recurrence of symptoms happened after 6 months of follow‐up.

## 1 CASE PRESENTATION

The patient was a 41‐year‐old male who presented to our rheumatology clinic with atrophic lesions of the neck and left ear and hyper‐pigmented plaques on the left thigh. He claimed that he developed bullous lesions on his neck, and left thigh about 2 weeks after injection of the second dose of COVID‐19 vaccination. When he was asked about the type of the injected vaccines, he reported that the first dose was Sinopharm BIBP COVID‐19 vaccine (BIBP‐CorV), and he voluntarily injected the second dose 1 month after the first one which was Oxford/AstraZeneca (ChAdOx1‐S [recombinant] vaccine) COVID‐19 vaccine. He did not inform the vaccination center about the time and type of the first injection. His past medical history, drug history, and familial and social history were unremarkable.

**FIGURE 1:**
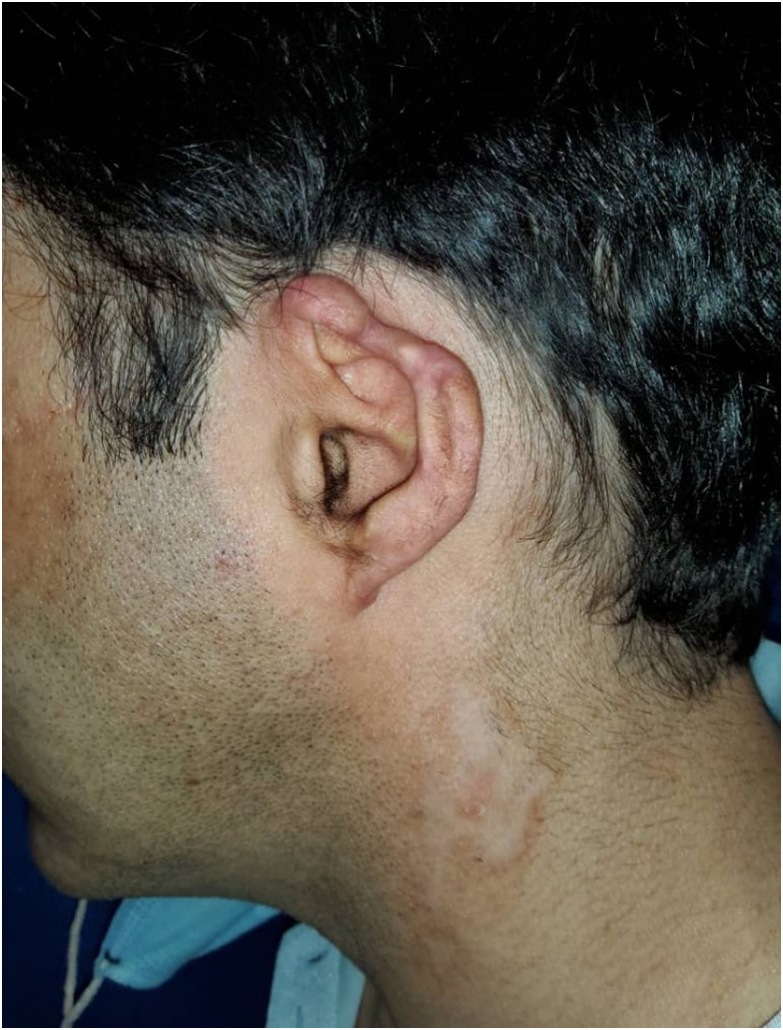
Showing the deformity and atrophy of the tip of the patient's left auricle, and an atrophic and depigmented patch on the lateral of the neck.

**FIGURE 2:**
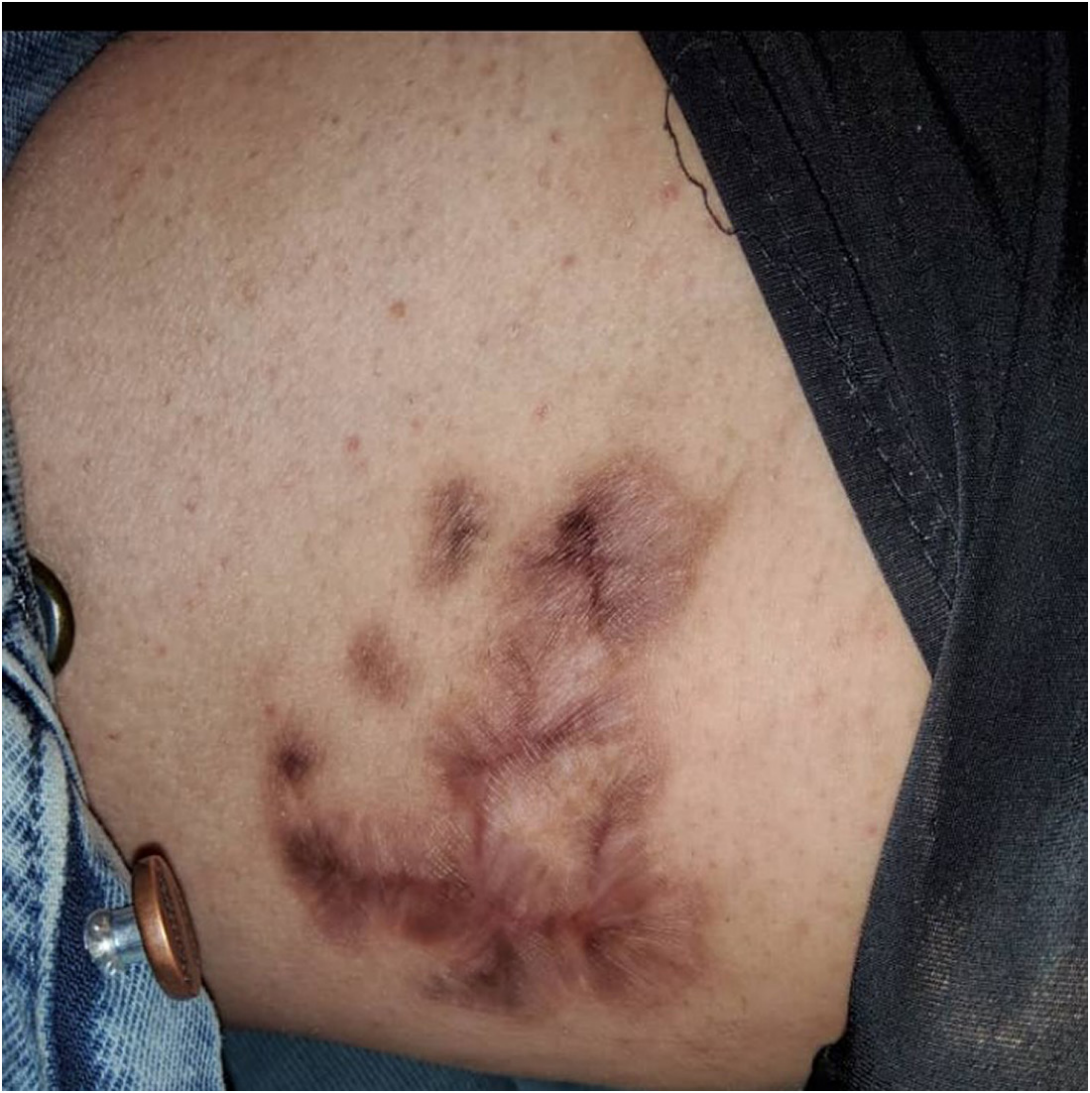
Showing the purple and pigmented plaques on the anterior of the left thigh.

On the meeting day, his vital signs, cardiovascular, respiratory, and neurological examinations were within normal limits except for cutaneous lesions. An atrophic and depigmented patch was observed on the lateral of the neck. The tip of his left auricle was atrophic and deformed (Figure 1). Purple and pigmented plaques were detected on the anterior of the left thigh (Figure 2). There were no signs or symptoms in favor of systemic inflammatory disease. Laboratory routine tests including cell blood count, renal and liver function tests, and thyroid function tests were all within normal limits.

A biopsy was taken from the thigh lesion which showed vasculitis of the small vessels with infiltration of inflammatory cells, mainly neutrophils in the vessel wall, and findings were compatible with leukocytoclastic vasculitis. Immunological tests were conducted to investigate possible systemic diseases and revealed negative results for antinuclear antibody (ANA), anti‐double‐stranded deoxyribonucleic acid (anti‐ds DNA) antibody, perinuclear and cytoplasmic anti‐neutrophil cytoplasmic antibody (P & C‐ANCA), Anti Ro, Anti La, angiotensin‐converting enzyme (ACE), C3, C4, CH50, and anti‐cyclic citrullinated peptide (anti‐CCP). Due to the absence of a systemic disease, the patient was treated with non‐steroidal anti‐inflammatory drugs and he was followed up monthly in a rheumatology clinic. There was no recurrence of the symptoms 6 months after the vaccine injection.

## 2 DISCUSSION

Cutaneous vasculitis (CV) is defined as an inflammatory disorder affecting the small vessels of the dermis. Clinical manifestations are urticarial, papules, plaques, palpable purpura, ulcers, nodules and livedo. Cutaneous vasculitis is classified based on the type of inflammatory infiltrations on the biopsy specimen, and the size of the involved vessels, etc. The most important subtypes are neutrophilic vasculitis, lymphocytic vasculitis, necrotizing vasculitis, thrombosing vasculitis, eosinophilic vasculitis, and granulomatous vasculitis. CV could be part of a systemic vasculitis or occur separately and be self‐limited. The most common etiologies are infections, autoimmune systemic disorders, malignancies, drugs, and vaccines. CV represents a self‐limited course in most cases. Systemic corticosteroids and immunosuppressive agents might be required in severe and systemic diseases.^1^ Different types of vasculitis have been reported as adverse events following immunization. Culprit vaccines are influenza, BCG, human papillomavirus, and hepatitis B vaccines.^2^ CV has been associated with both Coronavirus disease‐2019 (COVID‐19) and SARS‐CoV‐2 vaccines.^3^, ^4^ The inflammatory response triggered by the vaccine and immune complex deposition within the vessels wall of the dermis has been described as the cause of this adverse event. The responsible antigen for such an immune reaction might be the vaccine component encoding SARS‐CoV‐2 spike glycoprotein.^5^ In conclusion, LCV could be a possible adverse event of different SARS‐CoV‐2 vaccines. Clinicians and manufacturers should be aware of this adverse event.

## AUTHOR CONTRIBUTIONS

**Mehran Pournazari:** Conceptualization; supervision. **Shirin Assar:** Writing – review and editing. **Faraneh Farsad:** Data curation; investigation. **Dena Mohamadzadeh:** Writing – original draft.

## CONFLICT OF INTEREST STATEMENT

The authors declare that they have no competing interests.

## ETHICS APPROVAL AND CONSENT TO PARTICIPATE

Approval was not needed by the local Clinical Research Ethics Committee for case reports.

## CONSENT

Written informed consent was obtained from the patient for publication of this case report and any accompanying images. A copy of the written consent is available for review of the Editor‐in‐Chief of this journal.

## Data Availability

Data are available if requested.
